# Clinical Influence of Mandibular Flexure on Oral Rehabilitation: Narrative Review

**DOI:** 10.3390/ijerph192416748

**Published:** 2022-12-13

**Authors:** Eitan Mijiritsky, Maayan Shacham, Yuval Meilik, Michal Dekel-Steinkeller

**Affiliations:** 1 The Maurice and Gabriela Goldschleger School of Dental Medicine, Tel Aviv University, Tel Aviv 6997801, Israel; 2 Department of Otolaryngology, Head and Neck and Maxillofacial Surgery, Tel-Aviv Sourasky Medical Center, Sackler Faculty of Medicine, Tel Aviv 6139001, Israel; 3 School of Social Work, Ariel University, Ariel 40700, Israel

**Keywords:** mandibular flexure, prosthodontics, oral rehabilitation, mandibular movements, centric relation, vertical dimension of occlusion

## Abstract

Aim: The current paper aims to review mandibular flexure and its clinical implications in the field of oral rehabilitation. Mandibular flexure is a deformity of the mandible, which occurs during jaw movements. Methods and Materials: An electronic database search was conducted using the PRISM model, with a total of 49 articles included. Results: Mandibular flexure affects various stages of oral rehabilitation treatments. Effects of mandibular flexure are more significant in periodontal patients, and in implant-supported restorations, compared to natural teeth, due to differences in the force absorption by the periodontal ligament. Various adjustments must be made to the prosthodontic framework to enable long-term survival of the restorative treatments. Conclusions: Dental practitioners should pay attention to the following: (1) digital impressions are preferred over conventional; (2) mouth opening should be kept to a minimum (as possible, up to 10–20 mm) while also avoiding any anterior movements of the mandible (protrusion); (3) the number of abutment teeth should be kept to a minimum; (4) structures in the lower jaw should be splitted; (5) non-rigid connectors should be used to reduce the effort exerted; (6) in periodontal patients, the preference is for short-span restorations and non-rigid connectors; (7) in implant-supported restorations, it is preferable to divide the framework into two or three segments, utilizing rigid materials with a low elastic modulus. There is no agreement in the literature about the preferred location of the implants in the jaw.

## 1. Introduction

Mandibular flexure is a deformity in the mandible that occurs during mouth movements, mainly in mouth opening and in protrusive movements, and to a lesser extent during lateral working movements following the contraction of the masticatory muscles, mainly the inferior head of the lateral pterygoids which are held in the condylar process of the mandible on both sides. Mandibular flexure causes a reduction in the width of the mandible arch, where the bending is medial and is performed towards the symphysis structure in the frontal plane, together with the sagittal movement of the posterior aspect of the mandible [1,2,3]. The main cause of mandibular flexure is the bilateral contraction of the inferior head of the lateral pterygoid muscles that insert the condylar process of the mandible on each side, respectively [1,4,5].

There are various reports in the literature about the different aspects of the mandibular flexure phenomenon, affecting the understating of its importance in prosthodontic rehabilitations. The current review aims to review the existing literature about mandibular flexure in order to provide valuable information for dental practitioners involved in the field of prosthodontics.

According to previous studies, different parameters affect the mandibular flexure, such as: (i) vertical face aspect (people with vertically short faces (Brachyfacial) demonstrate the highest mandibular flexion values as opposed to people with vertically elongated faces (Dolychofacial)) [6,7]. (ii) Structure of the symphysis in the mandible, including its surface area, bone density, length, and labio-lingual thickness. A large surface area of the symphysis and high bone density both have a significant opposite effect on the level of mandibular flexure (flexion will be less significant when these values are higher). The length of the entire mandibular structure has a direct effect on the level of mandibular flexure (flexion is greater when the mandibular structure is longer), while the short length of the symphysis structure also has a direct effect on the level of mandibular flexure (flexion is greater when the symphysis structure is shorter). In addition, a small gonial angle was found to have a reduced but statistically proven effect on the level of mandibular flexion (the smaller the angle, the more significant the flexion) [2,8,9,10,11].

Additional parameters such as BMI, height, weight, maximum occlusal force (MOF), bruxism, tooth wear, and muscle pain have not been proven to have a statistically significant effect on mandibular flexion values [9,12]. In relation to MOF, the larger the values of height, weight, and BMI, the higher the values of MOF. However, these anthropometric variables did not influence mandibular flexure. Since these variables had a significant correlation with MOF, this would be an additional indication that MOF did not have a relation with mandibular flexure [12]. Other studies, such as the Chen DC et al. 2000 preliminary study on the mandibular flexure of dentate young volunteers, did not find a relation between these parameters and mandibular flexure and suggested more studies to be conducted evaluating muscle activity and its influence on mandibular flexure [9].

Regarding the relationship between age and level of mandibular flexure, even though in some studies it was demonstrated that age is not linked to the level of mandibular flexure in a statistically significantly matter, the study by Ebadian et al. demonstrated that old age is linked to an increase in the level of mandibular flexure. These findings were apparently due to a decrease in the number of teeth found more often in old age, which lead to a decrease in bone density (increased incidences of osteoporosis) and a short symphysis structure, both related to greater mandibular flexure values [13].

With relation to clinical prosthetic procedures, mandibular flexure seems to cause a reduction of a few tenths of a mm in the arch of the mandible. This reduction depends on various parameters, including jaw position during the measurement, the movements performed during the test (protrusion, opening, and lateral movements), the parameters that distinguish one mandible from another (bone density, bone size, and gender), and the clinical condition of the mandible (whether edentulous or not and whether there are any current prostheses) [14]. In addition, mandibular flexure may be involved in the potential mismatch between the prosthesis accuracy and abutment teeth or implants. The mismatch begins at the initial phase of impression-taking and reflects on the accuracy and fitting of the prosthesis to natural teeth and/or implants. When taking impressions, the patient is required to open his mouth, which activates the mastication muscles, causing mandibular flexure and therefore causing inaccuracies in the impressions. Evidence exists regarding the differences between conventional impression-taking and digital scanning [15,16,17]. From a biomechanical point of view, it can be said that unlike implants, in a natural dentition, the torsion efforts caused by mandibular flexion are mainly absorbed by the periodontal ligament but can also cause cracks (to a small extent) and consequent failures in restorations (especially porcelain, in the case of porcelain-fused-to-metal (PFM) restorations) or cementation. These effects are more significant in periodontally involved teeth and to a greater extent when it comes to patients with bruxism [5,18,19,20]. In implant-supported restorations, the two main parameters that contribute to the reduction in the efforts caused by mandibular flexion are the design of the restorative framework and distribution of implants in the mandible [18,21,22,23,24,25,26,27,28,29,30,31,32,33].

As noted above, mandibular flexure plays an important role in prosthodontics. It is a multifactorial phenomenon that results from various factors and should be considered in oral rehabilitation treatment procedures. Therefore, the current paper aims to examine the various parameters that influence mandibular flexure, including the various techniques and options available to minimize its negative consequences.

## 2. Materials and Methods

**Figure 1:**
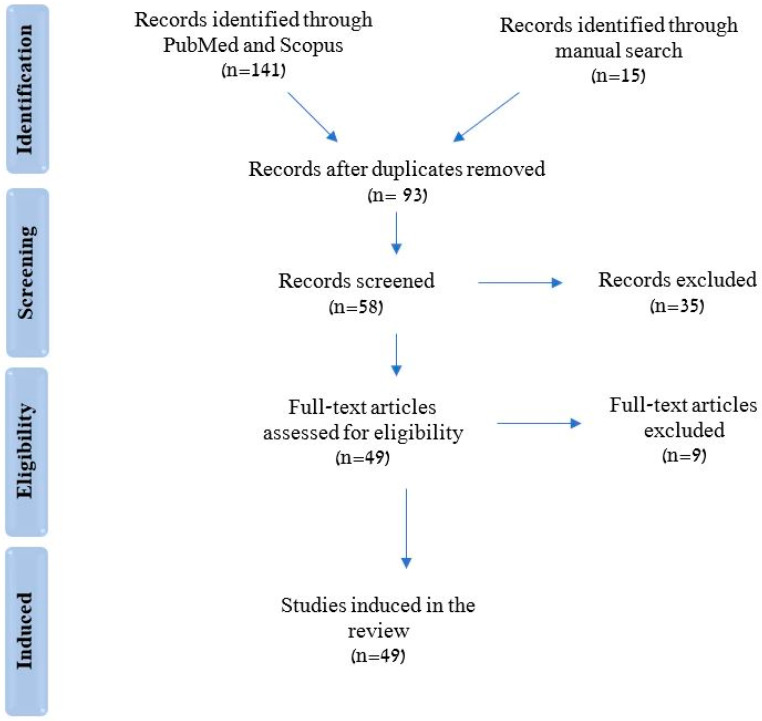
Flowchart depicting the selection process for included studies (PRISM flowchart).

Using main search engines (Pubmed and Scopus), along with Google Scholar, a narrative literature review was performed including papers published between 1960 and 2022 (see Figure 1). Keywords used were mandibular flexure, median mandibular flexure, mandibular deformation, oral rehabilitation, implant fixed prostheses, and superstructures, while filtering for English language, and according to predetermined inclusion and exclusion criteria.

Inclusion criteria: (i) studies or articles that review the action and forces of the mastication muscles and biomechanics of the mandibular movements for the purpose of adding relevant information; (ii) studies or articles reviewing mandibular flexure solely; (iii) studies or articles reviewing the clinical implications of the mandibular flexure on dental treatments in the field of prosthodontics only, including mathematical models and laboratory tests; and (iv) studies that use a control group with an equal representation of age among the subjects, the gender of the subjects, and so on.

Exclusion criteria: (i) studies or articles reviewing the mandibular flexure along with any other physiological or pathological problems; (ii) studies or articles that do not refer to mandibular flexure or the consequences of flexion on dental treatments in the field of prosthodontics; and (iii) studies or articles that review removable prosthodontic treatments.

## 3. Results

### 3.1. Mandibular Flexion Measurements

The mandibular flexion values vary within a range of a few tenths of a mm and depend on various parameters: the area in the mandible where the flexure was measured in the oral cavity or jaw, the type of movement performed during the measurement, parameters that differentiate one mandible from another (bone density, bone size, the person’s gender), and the clinical condition of the mandible (edentulous mandible or any prosthodontic treatments that were applied during the measurements.) The measurements range from 0 mm to 1.5 mm which were found in the article about protrusion movement by McDowell and Regli [14]; an average flexion of 0.4 mm while opening the mouth and an average of 0.5 mm while in protrusion (with a maximum of 1.5 mm) were recorded by splints that were attached to the occlusal surfaces of the second molars in the impressions and then were measured by gauges.

In Regli and Kelly’s article [19], the mandibular flexure was recorded upon opening of the mouth by taking impressions, and values of 0.03 mm were measured on average when testing the premolar area and 0.09 mm on average when testing the second molar area. Burch and Borchers [34], who measured the flexion by using a strain gauge, found that in the first molars the flexion was 0.61 mm while in protrusion, 0.438 mm while opening the mouth, 0.243 mm in right lateral movement, 0.257 mm in left lateral movement, and 0.006 mm as a minimum value in the individual tests.

Burch [35] measured the flexure with a strain gauge and found that the flexion value was equal to 0.432 mm on average in protrusion, 0.224 mm on average while opening the mouth, 0.112 mm on average in left lateral movement, and 0.105 mm in right lateral movement. Goodkind and Heringlake [4] measured 0.0768 mm on average while opening the mouth in the second molar area and 0.0316 mm when opening the mouth around the premolar area. This was measured by a gauge device called a federal test master dial micrometer in combination with taking impressions.

De Marco and Paine [36] documented the flexion of the occlusal surfaces in the first molars by using a gauge, with an average of 0.78 mm measured while opening the mouth (with a range of 0.6–1.5 mm). Gates and Nicholls [1] reported a flexion of 0–0.3 mm while opening the mouth, and 0.1–0.5 mm while in protrusion, which was measured by a linear variable differential transformer (LVDT) device and taking impressions. In Fischman’s article [5], an average of 0.07112 mm was measured while opening the mouth which was measured by gauges that were attached to the lingual surfaces of the first molars. Chen et al. [9] reported an average of 0.145 mm while opening the mouth, which was measured by using an LVDT device glued to the occlusal surfaces of the first molars. Canabarro and Shinkai [12] used digital scans of impressions and recorded the flexion around the first molars with an average of 0.146 mm while opening the mouth and 0.15 mm on average while in protrusion (see Table 1 for further information about the results of the current study).

Al-Shukhum et al. [37] documented the mandibular flexion by using a 3D finite element analysis: values of 0.8 mm were measured while the mouth was in a maximal opened position, 1.07 mm while in protrusion, 1.1 mm in right lateral movement, and 0.9 mm in left lateral movement. In another study by Al-Sukhun et al. [38], mandibular flexure was measured by using custom fabricated displacement transducers on implants in three different parameters. It was found that in the parameter of corporal approximation there was a flexion of 0.011–0.0578 mm, in the parameters of corporal rotation and in dorsoventral shear there was a flexion of 0.4–2.8 degrees. In addition, El-Sheikh et al. [39] recorded the mandibular flexure by using custom fabricated displacement transducers in three different parameters on implants. It was found that in the parameter of medial convergence there was a flexion of 0.015–0.042 mm when opening the mouth, 0.01–0.021 mm in lateral movements, and 0.018–0.053 mm in protrusion; in the parameter of corporal rotation, there was a flexion of 0.05–0.11 degrees upon opening the mouth, 0.03–0.08 degrees in lateral movements, and 0.03–0.15 degrees in protrusion; in the parameter of anteroposterior shear, there was a flexion of 0.038–0.093 mm upon opening the mouth, 0.028–0.056 mm in lateral movements, and 0.052–0.0103 mm in protrusion.

Asadzadeh et al. [40] reported a level of flexion while the mouth was open in different areas of the oral cavity by measuring with digital calipers. An average of 0.1894 mm was measured in the distal area of the second molars and 0.1671 mm in the distal area of the canines. Tulsani, Maiti, and Rupawat [41] measured the flexion with a digital vernier caliper, and an average flexion of 0.36375 mm was recorded while opening the mouth and 0.97375 mm while in protrusion. Wolf et al. [42], who measured the flexion while the mouth was open by taking impressions and inserting them into a digital measurer (three-dimensional evaluation), found that the flexion was 0.011 mm around the canines and 0.232 mm around the molars.

#### 3.1.1. Differences in Measurements According to the Area of Measurement

In measurements that were carried out in the frontal plane, it was found that the mandibular flexure had the highest values in the molar area, after that in the premolar area, and finally the lowest values were found in the canine area. The reason for this is the distance from the muscles of mastication, showing that the more posterior the area is in the mandible (closer to the mastication muscles), the more powerful the mandibular flexion [40].

Additional data that emerged from the study of Alvarez-Arenal et al. [11], who measured the flexion by a finite element analysis, were that while opening the mouth the flexion was most significant in the condyles, followed by the body of the mandible, and least significant in the symphyseal area which was actually the most stable area. In addition, during protrusion it was found that the flexion was mainly significant at the angle of the mandible [11].

#### 3.1.2. Differences in Measurements According to the Type of Movement Performed

According to Gates and Nicholls [1], the mandibular flexure was significant mainly in the performance of protrusion movements when compared to mouth opening. The reason for this is that while opening the mouth the anterior digastric muscles contract and do not contribute to the formation of flexion. The flexion starts from the moment the mouth opens, is escalated as the lateral pterygoid muscles are more active, and it is correspondingly greater when the mouth is more open (when comparing between maximal and minimal mouth opening). Similarly, lower values of mandibular flexion can be observed during lateral movements, in which only one muscle (lateral pterygoid) is active as opposed to protrusion in which both muscles are active at the same time. In addition, during retrusion movements an increase in the arch of the mandible has been recorded [11,35,37,41,42,43].

#### 3.1.3. Differences in Measurements According to Gender

A higher level of flexion was demonstrated in women compared to men, but at a level that was not statistically significant [9,12]. The mandibular flexure makes it possible to predict gender in 32–95.6% (depending on the various studies) of people by measuring various parameters on the mandible. The most accurate parameters for determination are the parameters that are measured in the upper part of the ramus above the curve (for example, measurements of the length and width of the ramus and measuring the maximal limit of mandibular flexion). Male mandibular verification is more accurate than female mandibular verification when comparing the two (i.e., women demonstrated higher deviations in the mandibular arch width measurements while opening the mouth) [42,44,45,46].

## 4. Discussion

The mandibular flexure has many clinical implications for various prosthodontic treatments, and therefore must be considered while making clinical manipulations and therapeutic adjustments in order to mitigate its consequences. Mandibular flexure has been measured in various studies over the years using different measurement techniques (intra- and extra-oral), in different areas of the mandible, in different mouth movements (retrusion, mouth opening, and lateral movements), in different measuring planes, and on natural teeth or on implants, and therefore it is difficult to compare them. In most of the studies documented in this review, the measurements were a few tenths of a mm and the difference between the measurements was due to the abovementioned parameters. Despite the differences between measurements, consistent conclusions emerge among the included studies, mostly because mandibular flexure is significant mainly in protrusion when compared to other jaw movements and is significantly higher mainly in the posterior areas of the jaw [11,35,37,41,42,43].

The mandibular flexure can affect the different stages of prosthodontic treatment, from impression-taking to adjusting the restoration and choosing the different treatment modalities.

### 4.1. The Effect of Mandibular Flexure on Impression-Taking

Taking impressions while opening the mouth wide and therefore causing maximum mandibular flexion may also affect the position of the central relation (CR) and create an inaccuracy in the occlusal adjustment of the prosthodontic treatment of the patient [17]. To minimize the damage in conventional impression-taking, the impression should be taken in a minimal opening of the mouth (as possible) and without anterior movements of the jaw (protrusion), so that the force exerted by the mastication muscles is reduced and the resulting mandibular flexion is minimal. During the hardening of the impressions, the dentist should avoid touching the patient’s mandible (pushing the mandible up or down). In addition, it is recommended to use vinyl polysiloxane (PVS), which demonstrates high dimensional stability and copies the best details compared to the other impression materials [5].

In a comparison between conventional impression-taking and digital impression-scanning, the digital scanning method was found to be more effective (with minimal values of the mandibular flexion) [15,16]. In the digital impression scan, there is no statistical significance difference of the mandibular flexion values when the mouth is partially or maximally opened. In addition, there is a conventional method of taking impressions by vinyl polysiloxane (PVS) and then digitally scanning them. In addition, with the usage of digital scans, the dentist must be well-acquainted with the digital system in order to use the systems properly [15,16].

### 4.2. The Effect of Mandibular Flexure on Teeth-Supported Prostheses

In tooth-supported restorations, the stress created by the mandibular flexure is absorbed in the periodontal ligament. The use of rigid connectors will limit teeth movement occurring during the flexion of the mandible and may increase the stress in the PDL. Long spans will also limit teeth movement, create increased stress in the PDL or in the prosthesis itself, and it may cause porcelain fractures or cement failures. In order to minimize the negative consequences of the mandibular flexion, several principles must be followed. According to Fischman’s studies from 1990 and 1976, the recommendations for prosthodontic treatments on natural dentition are to use a span as short as possible in permanent restorations, to avoid the use of porcelain when performing a long span, to split the structures into several centers on the mandible (as opposed to a full or very extensive prosthesis), and the use of non-rigid connectors that will reduce the applied efforts. In periodontal patients, it is also recommended to use a short span with non-rigid connectors [5,19,20].

### 4.3. The Effect of Mandibular Flexure on Implant-Supported Prostheses

In implant-supported restorations, several parameters should be considered: the division of the prosthodontic framework into segments, the material chosen to build the framework, and the position of the implants in the jaw.

The matter of dividing the prosthodontic framework into segments is controversial in the literature. According to seven different studies that were reviewed, it is recommended to divide the prosthodontic framework, as this will make it possible to reduce the tension created by the force of mandibular flexion. When dividing the skeleton into two segments, it is recommended to divide it along the symphyseal line of the mandible. Such division in the midline allows the most ideal way to create the natural biomechanical behavior of the mandible and reduce the tension on the implants [18,21,23,24,25,26,47]. On the other hand, according to two other studies reviewed, it was found that it is preferable not to divide the prosthodontic framework, as it provides a more natural biomechanical environment for the mandible and lower peri-implant bone stress. Nevertheless, in these studies, it was found that when dividing the framework there is a preference for dividing it into two segments in the midline rather than three segments; this was also found in previous studies which supported the division of the skeleton [24,48].

Regarding the material chosen to be used to build the framework, according to two different studies that were reviewed [10,27], it was found that a high elastic modulus of the material used to build the prosthodontic framework makes it possible to reduce the intensity of the mandibular flexion through the greater absorption of efforts. In a third study that was reviewed (Marin et al.), it was found that materials with a low elastic modulus (combined with the division of the framework into segments) make it possible to absorb the efforts applied during mandibular flexion more efficiently [10,21,27].

The position of the implants in the jaw and its effect on the mandibular flexion is also much disputed in the literature. Four studies favored the mesial placement of the implants, claiming that this position allows the jaw to have greater flexibility along with lowering pressing efforts on the implants. On the other hand, three studies were in favor of placing the implants distally, because according to their study the peri-implant bone stress is lower in this position [18,24,25,28,29,30,31,32,33]. The mandibular elastic deformation is modified by the insertion of different types of fixed implant-supported restorations, creating a different influence on the mandibular flexure. Deformation of the implant- restored mandible is significantly affected by at least two factors: the position of the implants and the design of the prosthetic superstructure. There is a controversy in the literature regarding the location and position of the implants in the mandible. Some preferred more mesially located implants, as in the work of Zarone F. et al. [18], demonstrating the overall flexibility of the implant-restored mandible is significantly increased as the more distal implant supports of the prosthesis were more mesially located, creating less stress on the peri-implant bone. Others preferred more distally located implants, as in the work of Nokar et al. [25], demonstrating a higher peri-implant bone stress in the more mesially located implants. The lack of consensus is probably due to the large number of parameters influencing the mandibular flexure in the implant-supported restorations along with the position of the implants and the design of the framework, such as the material used, the type of prosthesis (splinted or non-splinted), the type of movement (lateral movement vs. protrusion or opening), etc.

In addition, the structure of the mandible (the surface area, the dimensions of the symphysis, and the size of the gonial angle), together with the gender of the person (in women a greater mandibular flexion was demonstrated), and the structure of the face in the vertical dimension are parameters that may be considered when calculating the placement of the implants and while evaluating the bone for the prosthodontic treatment, because they all have great clinical significance on the level of mandibular flexion. Parameters such as BMI, height, weight, maximum occlusal force (MOF), bruxism, tooth wear, and muscle pain have no statistical significance on the value of mandibular flexion [2,9,12,13].

## 5. Conclusions

Based on the results of the current review, we suggest the following recommendations for prosthetic treatment while considering the mandibular flexure phenomenon.

### 5.1. Taking Impressions

- There is a preference for using digital scanning instead of conventional impression-taking, which often also allows the avoidance of maximal mouth opening.
- If conventional impressions are taken, the impression should be made with minimal mouth opening (as possible, up to 10–20 mm) while also avoiding any anterior movements of the mandible (protrusion) and avoiding contact with the patient’s mandible during the hardening process of the material. In addition, it is recommended to use vinyl polysiloxane (PVS).

### 5.2. Dental Prosthesis on Natural Dentition

- It is recommended to reduce the use of porcelain in cases of extensive prosthodontic restorations.
- Splitting structures into several sections when reconstructing the lower jaw (as opposed to a full or very extensive prosthodontic restoration) and the use of non-rigid connectors that reduce the applied efforts are recommended.
- In periodontal patients, usage of short span and non-rigid connectors are recommended.

### 5.3. Implant-Supported Prostheses

- There is a preference for dividing the implant-supported prosthodontic framework into 2 or 3 segments.
- There is a preference for using rigid materials with a high elastic modulus.
- There is no consensus regarding the distribution of the implants in the jaw (mesial or distal implant placement).

## Figures and Tables

**Table 1:** Summary of studies included in the current narrative review.

Ref. Num.	Title and Authors	Year of Publish	Article Type	Method Testing the MF ^2^	IO/EO ^1^	Type of Rehabilitation	Num. of Subjects Tested	Main Results
1	Evaluation of mandibular arch width change, Gates GN, Nicholls JI	1981	Clinical trial	LVDT and casts	EO	Natural dentition	10 males	0–0.3 mm of MF in protrusion, 0.1–0.5 mm of MF in mouth opening
2	Stress and strain in the mandibular symphysis of primates: a test of competing hypotheses, Hylander WL	1984	Clinical trial	Strain gauges	EO	Natural dentition	6 Macaca fascicularis	Establishing various hypotheses about symphyseal stress and strain
3	Biomechanics of the mandible, Van Eijden TM	2000	A systematic review	The behavior of the mandibular bone in response to external loading	IO and EO	Natural dentition	Varies based on the article that was reviewed	Establishing various patterns of stress and strain of the mandible
4	Mandibular flexure in opening and closing movements, Goodkind RJ, Heringlake CB	1973	Clinical trial	Federal test master dial micrometer (gauges) and impressions	IO	Natural dentition	40 (20 males and 20 females)	0.0768/0.0316 mm of MF in mouth opening in different landmarks
5	The rotational aspect of mandibular flexure, Fischman B	1990	Clinical trial	Gauges	IO	Natural dentition	10 (4 males and 6 females)	0.07112 mm of MF in mouth opening
6	Occlusal force, electromyographic activity of masticatory muscles and mandibular flexure of subjects with different facial types, Custodio W, Gomes SGF, Faot F, Garcia RCMR, Del Bel Cury AA	2011	Clinical trial	Analyzing scans of impressions	EO	Natural dentition	78 (39 males and 39 females)	Brachyfacial subjects had the highest MF records, followed by the mesofacial and the dolychofacial subjects
7	Median mandibular flexure at different mouth opening and its relation to different facial types: A prospective clinical study, Prasad M, Hussain MZ, Shetty SK, et al.	2013	Clinical trial	Analyzing scans of impressions	EO	Natural dentition	60 (males and females)	Brachyfacial subjects had the highest MF records, followed by the mesofacial and the dolychofacial subjects
8	Maximum occlusal force and medial mandibular flexure in relation to vertical facial pattern: a cross-sectional study, Shinkai RS, Lazzari FL, Canabarro SA, et al.	2007	Clinical trial	Analyzing scans of impressions	EO	Natural dentition	51 (24 males and 27 females)	No differences between different facial types in mandibular flexure, BMI is not a significant covariate for MF
9	Contributing factors of mandibular deformation during mouth opening, Chen DC, Lai YL, Chi LY, Lee SY	2000	Clinical trial	LVDT	IO	Natural dentition	62 (28 males and 34 females)	0.145 mm of MF in mouth opening, determining the influence of different variants on MF (gender, symphyseal area, width and height, bone density, gonial angle)
10	Numerical study of the influence of material parameters on the mechanical behaviour of a rehabilitated edentulous mandible, Favot L-M, Berry-Kromer V, Haboussi M, Thiebaud F, Zineb T Ben	2014	Clinical trial	Finite element model	EO	Edentulus mandible with complete prosthetic rehabilitation on 4 implants	3 cortical bone thicknesses with 4 different materials (Zr, Ti, Au, and NiTi)	MF decreased with an increase in the cortical bone thickness and stiffness of the prosthetic framework’s material
11	A jaw model for the study of the mandibular flexure taking into account the anisotropy of the bone, Alvarez-Arenal A, Lasheras FS, Fernández EM, González I	2009	Clinical trial	Finite element model	EO	Edentulus mandible	8 different bone mineral density with 3 different mandibular movements	Determining the influence of bone mineral density on MF, the response of different areas in the mandible following MF in protrusion and mouth opening and measurements of the MF
12	Medial mandibular flexure and maximum occlusal force in dentate adults, Canabarro S de A, Shinkai RSA	2006	Clinical trial	Analyzing scans of impressions	EO	Natural dentition	80 (40 males and 40 females)	Determining the influence of different variants on MF (MOF, gender, weight, height, BMI, and age) and evaluating MF values (0.146 mm for mouth opening and 0.15 mm for protrusion)
13	Assessment of the relationship between maximum occlusal force and median mandibular flexure in adults: A clinical trial study, Ebadian B, Abolhasani M, Heidarpour A, Ziaei M, Jowkar M	2020	Clinical trial	Digital calipers	EO	Natural dentition	90 (45 males and 45 females)	Determining the influence of different variants on MF (MOF, gender, age, and BMI)
14	A quantitative analysis of the decrease in width of the mandibular arch during forced movements of the mandible, McDowell JA, Regli CP	1961	Clinical trial	Splints and gauges	EO	Natural dentition	20 males and females	0.4 mm of MF in mouth opening and 0.5 mm of MF in protrusion
15	Mandibular flexure associated with muscle force applied in the retruded axis position, Omar R, Wise MD	1981	Clinical trial	Carrying vehicle and dial gauge indicator	IO	Natural dentition	10 males and females	A ”closed mouth” impression technique (or a minimal opening of the mouth) gives the best results for conventional impression’s technique
16	Elastic deformation of the mandibular jaw revisited—a clinical comparison between digital and conventional impressions using a reference, Schmidt A, Klussmann L, Schlenz MA, Wöstmann B	2021	Clinical trial	Intraoral scanner and conventional impression	IO	Natural dentition	50 males and females	No mandibular deformation could be detected during mouth opening with regard to the digital impressions
17	Reliability of a digital image method for measuring medial mandibular flexure in dentate subjects, Shinkai RSA, Canabarro S de A, Schmidt CB, Sartori EA	2004	Clinical trial	Analyzing scans of impressions and digital calipers	EO	Natural dentition	7 males and females	Records of MF with digital images have less distortions and are more accurate
18	Mandibular flexure and stress build-up in mandibular full-arch fixed prostheses supported by osseointegrated implants, Zarone F, Apicella A, Nicolais L, Aversa R, Sorrentino R	2003	Clinical trial	Finite element model	EO	Edentulous mandible with 6 types of implant-supported prosthetics systems	1 male	MF causes a significant amount of stress in the more distal implants and the superstructures, and division of the superstructure at the level of the symphysis is recommended
19	The phenomenon of decreased mandibular arch width in opening movements, Regli CP, Kelly EK	1967	Clinical trial	Impressions	EO	Natural dentition	62 males and females	0.03/0.09 mm of MF in mouth opening in different landmarks
20	The influence of fixed splints on mandibular flexure, Fischman BM	1976	Clinical trial	Casts and calipers	EO	Natural dentition	1 female	MF reduces when fixed splints are present in the mouth
21	Split-Framework in Mandibular Implant-Supported Prosthesis, Marin DOM, Dias K de C, Paleari AG, Pero AC, Arioli Filho JN, Compagnoni MA	2015	A case report	Satisfactory clinical outcomes of the procedure	IO	Edentulous mandible, with 6 implants and a fixed full prosthesis	1 male	The use of split framework compensates MF and reduces the risk of loss of the posterior implants or screw loosening with acceptable patient comfort
22	In vivo mandibular elastic deformation during clenching on pivots, Jiang T, Ai M	2002	Clinical trial	Charge coupled device cameras and an image analyzing system	IO	Natural dentition	4 males	The influence of the MF on the connected prosthesis is negligible in the case of the natural tooth-supported long span bridge, but should probably be considered in the case of the implant-supported bridge
23	Implant-supported mandibular splinting affects temporomandibular joint biomechanics, Zaugg B, Hämmerle CHF, Palla S, Gallo LM	2012	Clinical trial	Digital analyzing of casts	EO	2 implants and a natural anterior dentition for each subject	6 (4 males and 2 females)	Transversal splinting reduces MF during jaw opening–closing and the distance between lateral condylar poles (changes loading patterns of the TMJ structures)
24	Mandibular flexure and peri-implant bone stress distribution on an implant-supported fixed full-arch mandibular prosthesis: 3D finite element analysis, Martin-Fernandez E, Gonzalez-Gonzalez I, deLlanos-Lanchares H, Mauvezin-Quevedo MA, Brizuela-Velasco A, Alvarez-Arenal A	2018	Clinical trial	Finite element model	EO	Edentulous mandibles with 6 implants and full rehabilitation	3 different FNE models with 3 types of frameworks	The undivided framework provides the best biomechanical environment during mandibular protrusion and opening. Protrusion movement increases the peri-implant bone stress. The most mesial implants have the lowest biomechanical risk
25	The effect of superstructure design on stress distribution in peri-implant bone during mandibular flexure, Nokar S, Naini RB	2010	Clinical trial	Finite element model	EO	Edentulous mandibles with 6 implants and full rehabilitation	1 FNE with 2 types of frameworks	MF should take into consideration in the design of implant-supported fixed partial dentures in the mandible
26	Three-dimensional finite element analysis of the effect of 1-piece superstructure on mandibular flexure, Naini RB, Nokar S	2009	Clinical trial	Finite element model	EO	Edentulous mandibles with 5 implants and full rehabilitation	1 FNE	One-piece implant-supported superstructure restricted mandibular deformation to almost half of the amount observed in unrestricted mandibular models of previous studies
27	Clinical methods for evaluating implant framework fit, Kan JYK, Rungcharassaeng K, Bohsali K, Goodacre CJ, Lang BR	1999	A systematic review	Clinical and radiographics evaluation	IO	Fixed implant-supported framework	Varies between the articles reviewed	Implant components and bone appear to tolerate a degree of misfit without adverse biomechanical problems, improving clinical techniques may be relied on to optimize fit or compensate for misfit
28	Stress analysis in edentulous mandibular bone supporting implant-retained 1-piece or multiple superstructures, Yokoyama S, Wakabayashi N, Shiota M, Ohyama T	2005	Clinical trial	Finite element model	EO	Edentulous mandibles with 8 implants and full rehabilitation	1 FNE 3 types of frameworks	The unseparated superstructures were more effective in relieving stress of concentration in the edentulous mandibular bone than the separated superstructures
29	Effect of abutment’s height and framework alloy on the load distribution of mandibular cantilevered implant-supported prosthesis, Suedam V, Capello Souza EA, Moura MS, Jacques LB, Rubo JH	2009	Clinical trial	Strain gauges	EO	Implant-supported rehabilitation (no mention for dentition)	2 types of alloys, 3 frameworks	Abutment’s height and framework alloy influence the deformation of abutments of mandibular cantilevered implant-supported prosthesis
30	The Effect of Mandibular Flexure on Stress Distribution in the All-on-4 Treated Edentulous Mandible: A Comparative Finite-Element Study Based on Mechanostat Theory, Shahriari S, Parandakh A, Khani M-M, et al.	2019	Clinical trial	Finite element model	EO	Edentulous mandible with 4 implants, no mention for rehabilitation above the implants	2 FNE	Use of tilted implants in the treatment of edentulous mandible would reduce the probability of bone loss in vulnerable parts of the osseous tissue surrounding dental implants
31	Comparison of tilted versus nontilted implant-supported prosthetic designs for the restoration of the edentuous mandible: a biomechanical study, Bellini CM, Romeo D, Galbusera F, et al.	2009	Clinical trial	Finite element model	EO	2 edentulous mandibles with 4 implants and full rehabilitation, 1 edentulous mandible with 5 implants and full rehabilitation	3 models	Higher values for compressive stress were predicted near the cervical area of the distal implant in the tilted model
32	Influence of implant framework and mandibular flexure on the strain distribution on a Kennedy class II mandible restored with a long-span implant fixed restoration: a pilot study, Law C, Bennani V, Lyons K, Swain M	2014	Clinical trial	Strain gauges	EO	Partially edentulous mandible with 2 implants and fixed splints	1 CT scan, 3 frameworks	When frameworks were placed and a unilateral load applied, compression was detected on the mesial and buccal aspect of the mesial implant with all 3 frameworks. The amount of strain recorded was higher than that recorded without any framework in place
33	Use of interimplant displacement to measure mandibular distortion during jaw movements in humans, Horiuchi M, Ichikawa T, Noda M, Matsumoto N	1997	Clinical trial	A micromagnetic sensor	IO	2 implants and 2 fabricated crowns for each subject, no mention for dentition	4 (1 male and 3 females)	The distal implant deviated to the lingual side relative to the mesial implant and the deviation with jaw protrusion was larger than that with opening movement
34	Mandibular flexure and dental implants: a case report, De Oliveira RM, Emtiaz S	2000	A case report	Results are based on clinical findings	IO	Edentulous mandibles with 6 implants and full rehabilitation	1 male	In situations where implants are placed as far distally as the molar areas in the mandible, functional distortion and the resultant potential force transmission between fixtures could be of major significance for implant-supported restorations
35	Influence of mandibular superstructure shape on implant stresses during simulated posterior biting, Korioth TWP, Johann AR	1999	Clinical trial	Finite element model	EO	5 implants and a rehabilitation supported on top of them, no mention for dentition	1 FNE	Simulated implant abutment stresses may be significantly affected by the shape of the prosthetic superstructure, by diverse mandibular loading conditions, and to a lesser extent, by the prosthetic material properties
36	Method for study of mandibular arch width change, Burch JG, Borchers G	1970	Clinical trial	Strain gauges	IO	Natural dentition	10 males and females	0.61 mm of MF in protrusion, 0.438 mm of MF in mouth opening, 0.243/0.257 mm of MF in lateral movements
37	Patterns of change in human mandibular arch width during jaw excursions, Burch JG	1972	Clinical trial	Strain gauges	IO	Natural dentition	25 males and females	0.432 mm of MF in protrusion, 0.224 mm of MF in mouth opening, 0.112/0.105 mm of MF in lateral movements
38	Mandibular dimensional change, De Marco TJ, Paine S	1974	Clinical trial	Gauges	IO	Natural dentition	25 (7 males and 18 females)	0.78 mm of MF in mouth opening
39	Biomechanics of the mandible: Part II. Development of a 3-dimensional finite element model to study mandibular functional deformation in subjects treated with dental implants, Al-Sukhun J, Kelleway J	2007	Clinical trial	Finite element model	EO	Edentulous subjects with 2 implants for each subject, no mention for rehabilitation above the implants	12 females	0.8 mm of MF in mouth opening, 1.07 mm of MF in protrusion, 1.1/0.9 mm of MF in lateral movements
40	Biomechanics of the mandible part I: measurement of mandibular functional deformation using custom-fabricated displacement transducers, Al-Sukhun J, Helenius M, Lindqvist C, Kelleway J	2006	Clinical trial	Custom fabricated displacement transducers	IO	Edentulous subjects with 2 implants for each subject, no mention for rehabilitation above the implants	12 females	Testing MF in different jaw deformation types (0.011–0.0578 mm of MF in corporal approximation and 0.4–2.8° of MF in corporal rotation and dorsoventral shear)
41	Midline mandibular deformation during nonmasticatory functional movements in edentulous subjects with dental implants, El-Sheikh AM, Abdel-Latif HH, Howell PGT, Hobkirk JA	2007	Clinical trial	Custom fabricated displacement transducers	EO	Edentulous subjects with 2 implants for each subject, no mention for rehabilitation above the implants	5 (1 male and 4 females)	Testing MF in 3 different jaw deformation types and in 3 different jaw movements
42	Mandibular width and length deformation during mouth opening in female dental students, Asadzadeh N, Madani AS, Mirmortazavi A, Sabooni MR, Shibani V	2012	Clinical trial	Digital calipers	IO	Natural dentition	35 females	0.1894/0.1671 mm of MF in mouth opening in different landmarks
43	Evaluation of change in mandibular width during maximum mouth opening and protrusion, Tulsani M, Maiti S, Rupawat D	2019	Clinical trial	Digital vernier calipers	IO	Natural dentition	140 males and females	0.36375 mm of MF in mouth opening and 0.97375 mm of MF in protrusion
44	Three-dimensional evaluation of mandibular deformation during mouth opening, Wolf L, Bergauer B, Adler W, Wichmann M, Matta RE	2019	Clinical trial	Analyzing scans of impressions	EO	Natural dentition	40 (20 males and 20 females)	0.011/0.232 mm of MF in mouth opening in different landmarks, evaluation of the MF by gender
45	Functional mandibular deformation in edentulous subjects treated with dental implants, Abdel-Latif HH, Hobkirk JA, Kelleway JP	2000	Clinical trial	Custom fabricated strain gauges	EO	Edentulous subjects with 2 implants for each subject, no mention for rehabilitation above the implants	6 males and females	Testing MF in 3 different jaw deformation types (medial convergence, corporal rotation, and dorsoventral shear)
46	Sex determination from the mandibular ramus flexure of Koreans by discrimination function analysis using three-dimensional mandible models, Lin C, Jiao B, Liu S, et al.	2014	Clinical trial	Analyzing of 3D models of the mandible	EO	Natural dentition	240 cranial CT scans (120 males and 120 females)	Upper ramus above flexure holds larger potential than the mandibular ramus flexure itself to predict sexes
47	Mandibular ramus flexure and gonial eversion as morphologic indicators of sex, Kemkes-Grottenthaler A, Löbig F, Stock F	2002	Clinical trial	Observers on forensic samples and archeological provenance samples	EO	No mention in the text	233 (153 forensic and 80 archaeological provenance)	66% of accuracy for males and 32% accuracy for females
48	Predictive accuracy of sexing the mandible by ramus flexure, Balci Y, Yavuz MF, Cağdir S	2005	Clinical trial	Observers on forensic samples	EO	Natural dentition, 35 of subjects were with excessive tooth loss	120 forensic (95 males and 25 females)	95.6% of accuracy for males and 70.6% accuracy for females
49	Split-frame implant prosthesis designed to compensate for mandibular flexure: a clinical report, Paez CY, Barco T, Roushdy S, Andres C	2003	A case report	Satisfactory clinical outcomes of the procedure	IO	Edentulous mandible, with 8 implants and a fixed full prosthesis	1 male	Separating the prosthesis at the midline can relieve stress and strain during MF

^1^ Intra-oral/Extra-oral; ^2^ Mandibular flexure.

## Data Availability

Not applicable.
